# Dexamethasone and postoperative hyperglycemia in diabetics undergoing elective hip or knee arthroplasty: a case control study in 238 patients

**DOI:** 10.1186/s13037-018-0178-9

**Published:** 2018-11-05

**Authors:** Robert S. O’Connell, Bryce N. Clinger, Erin E. Donahue, Francesco S. Celi, Gregory J. Golladay

**Affiliations:** 1 Department of Orthopaedic Surgery, Virginia Commonwealth University, P.O. Box 980153, Richmond, Virginia 23298 USA; 2 School of Medicine, Virginia Commonwealth University, 1201 E Marshall St,, Richmond, Virginia 23298 USA; 3 Department of Biostatistics, School of Medicine, Virginia Commonwealth University, P.O. Box 980032, Richmond, Virginia 23298 USA; 4 Division of Endocrinology Diabetes and Metabolism, Department of Internal Medicine, Virginia Commonwealth University, 1101 East Marshall Street, Sanger Hall, PO Box 980111, Richmond, Virginia 23298 USA; 5 Department of Orthopaedic Surgery, Virginia Commonwealth University, P.O. Box 980153, Richmond, Virginia 23298 USA

**Keywords:** Total joint arthroplasty, Hyperglycemia, Dexamethasone, Optimization, Prosthetic joint infection

## Abstract

**Background:**

Dexamethasone has been routinely used in the pre-operative setting to enhance analgesia and decrease the incidence of nausea and vomiting in patients undergoing primary arthroplasty. However, dexamethasone has the potential to increase blood glucose levels postoperatively, which is a known risk factor for complications after total joint arthroplasty. The aim of this study was to analyze the effect of dexamethasone administration on post-operative blood glucose levels in diabetic patients after primary hip and knee arthroplasty.

**Methods:**

This study was a retrospective review of 238 diabetic patients who underwent primary hip and knee arthroplasty between May 1, 2014 and September 30, 2016 at a single urban academic medical center. A total of 77 patients (32.4%) received dexamethasone and 161 (67.7%) did not. Oral hyperglycemic agents were held during the inpatient stay and blood glucose was controlled either with sliding scale insulin or home insulin regimens were continued. All analyses were adjusted for age, BMI, gender, type of diabetes, pre-operative diabetic medication, type of surgical procedure, and pre-operative HgbA1c level. The primary outcome was post-operative hyperglycemia within 72 h of surgery defined as any blood glucose level greater than or equal to 200 mg/dL.

**Results:**

Post-operative hyperglycemia was observed in 17.1 and 20.6% of the measurements during the first 24 and 72 h respectively. After controlling for confounding variables, patients who received dexamethasone had 4.07 (95% CI: 2.46, 6.72) and 3.08 (95% CI: 2.34, 4.04) higher odds of post-operative hyperglycemia in the first 24 and 72 h respectively.

**Conclusions:**

Dexamethasone administration in diabetic patients undergoing primary arthroplasty increases post-operative hyperglycemia during the first 24 and 72 h. While our data did not investigate causation, dexamethasone use in this patient population should be thoughtfully considered, as post-operative hyperglycemia is a known risk factor for complications.

## Background

The recent introduction of bundled payment initiatives in lower extremity arthroplasty has resulted in increased attention to perioperative optimization and accelerated rehabilitation protocols [1]. Multimodal analgesia has been shown to be effective for pain management while decreasing opioid-induced side effects and facilitating early mobilization. However, post-operative nausea and vomiting still occurs in up to 86% of patients after hip and knee arthroplasty [2]. Dexamethasone has been shown to be effective in mitigating post-operative nausea and vomiting; furthermore, total joint arthroplasty prospective randomized trials have shown it can enhance analgesic effects, improve outcomes, and decrease length of stay (LOS) [2–8].

However, dexamethasone may cause a transient increase in blood glucose levels, and poor perioperative glycemic control is a risk factor for postoperative complications including infection [9–13]. The purpose of this study was to assess the effect of dexamethasone administration on post-operative blood glucose levels in the first 72 h after primary hip or knee arthroplasty in diabetic patients. We hypothesized that dexamethasone administration in diabetic patients after primary hip and knee arthroplasty would increase the risk of post-operative hyperglycemia.

## Methods

After obtaining institutional review board approval, a retrospective review was conducted of all diabetic patients who underwent primary total hip arthroplasty (THA), conversion total hip arthroplasty, total knee arthroplasty (TKA), and unicondylar knee arthroplasty (UKA) between May 1, 2014 and September 30, 2016 at an urban academic medical center. Patients were identified using a search of the Current Procedural Terminology (CPT) codes in our hospital’s billing database using the following codes: 27447 (TKA), 27,130 (THA), 27,132 (conversion THA), and 27,446 (UKA). This search was filtered to identify patients with a known diagnosis of diabetes (CTP code 250.x) including both type I and type II and patients with diet controlled diabetes. Exclusion criteria included revision arthroplasty, age < 18, pregnant patients, prisoners, previous infection in the affected joint, or tumor in the affected joint. We collected demographic information and clinical data points including body mass index (BMI), pre-operative hemoglobin A1C level (HgbA1C), pre-operative diabetic medication regimen, procedure performed, dexamethasone administration, length of stay, and all post-operative blood glucose levels for the first 72 h. The patients were then subdivided into two groups based on dexamethasone administration. If given, dexamethasone was administered in the perioperative holding area by the anesthesia team. Dexamethasone dosage and administration varied according to individual surgeon and anesthesiologist preferences.

At our institution, there is a pathway for elective arthroplasty to help minimize complications and maximize patient satisfaction and outcomes. In the pre-operative setting this includes strict selection criteria including a BMI < 40, HgbA1c < 8%, albumin > 3.5, and hemoglobin > 12 mg/dL as well as pre-operative optimization as indicated by individual comorbidities. All patients undergo a pre-operative anesthesia consult as well as attend a joint replacement class. They are encouraged to hydrate prior to the day of surgery and are made NPO at midnight prior to surgery with clear fluids allowed until 2 h before surgery. At the time of this study, there was no standardized preoperative carbohydrate drink. Peri-operatively, patients are offered spinal anesthesia unless there is a contraindication. They are given an appropriately dosed multimodal pain regimen including acetaminophen, gabapentin, and celecoxib and a peri-articular joint injection including toradol, clonidine, epinephrine, and ropivacaine. Intra-operative fluid resuscitation is monitored at the discretion of the anesthesiologist. Post-operatively patients were ambulated postoperative day zero if medically stable and were discharged once pain and institutional mobility criteria for safe discharge were met.

Blood glucose levels were obtained per standard protocol for all diabetic patients on the orthopedic floor post-operatively three times a day before meals and once before bed resulting in four potential data points each hospital day. However, preoperative (day of surgery) blood glucose levels are not routinely collected at our institution. All patients had their oral hyperglycemic agents held during their inpatient stay and were given a diabetic diet. Patients were either placed on their home insulin regimen or were given sliding scale rapid acting insulin based on measured glucose levels, however there was no standardized sliding scale or insulin regimen used. The primary outcome was post-operative hyperglycemia defined as any blood glucose concentration greater than or equal to 200 mg/dL within the first 72 h after surgery. The secondary outcomes of the study included post-operative hyperglycemia defined as any blood glucose concentration greater than or equal to 200 mg/dL within the first 24 h after surgery, post-operative length of stay (defined in hours), the relationship between dose of dexamethasone and post-operative blood glucose levels of diabetic patients in the first 72 h, and mean blood glucose.

Continuous variables were summarized by means and standard deviations, whereas categorical variables were summarized by frequencies and percentages. Two-sample t-tests, Wilcoxon tests and Pearson chi-square tests were conducted to determine if there were differences between the dexamethasone and non-dexamethasone groups. In order to assess the effect of dexamethasone use on post-operative blood glucose levels, with the primary objective considering the first 72 h after surgery and a secondary outcome considering the first 24 h after surgery, binomial logistic regression was used. Odds ratios and their confidence intervals were reported. Further, the relationship between dexamethasone use and length of stay was assessed by a multiple linear regression. Differences and their confidence intervals as well as *p*-values were reported. Additionally, a longitudinal general linear model assessed the relationship between dexamethasone and mean blood glucose concentration. All analyses were adjusted for age, BMI, gender, type of diabetes, pre-operative diabetic medication, type of surgical procedure, and pre-operative HgbA1c level. All statistical analyses were conducted in SAS 9.4 (Cary) with a significance level of 0.05.

## Results

A total of 238 patients were identified and met our inclusion criteria. The demographic and clinical characteristics are summarized in Table 1. Seventy-seven patients (32.4%) received dexamethasone perioperatively, in any dose, while 161 patients (67.7%) did not receive dexamethasone perioperatively. Dexamethasone doses were 4, 5, 8, or 10 mg, with 5 (6.5%), 6 (7.8%), 5 (6.5%), and 61 (79.2%) patients in each group, respectively. On average, blood glucose levels were elevated in 17.1 and 20.6% of measurements in the first 24 and 72 h respectively. Of the 77 patients in the study who received dexamethasone, 41 (53.2%) and 31 (40%) developed hyperglycemia in the first 72 and 24 h after surgery respectively. Of the 161 subjects who did not receive dexamethasone, 62 (38.5%) and 30 (18.6%) developed hyperglycemia in the first 72 and 24 h after surgery respectively. Table 2 displays the patient characteristics by dexamethasone group. Patients in the non-dexamethasone group were younger (*P* = 0.021) and had a higher BMI (*P* = 0.031). Further, the two groups were significantly different by type of diabetic medication (*P* = 0.020) and procedure (*P*≤0.001).

**Table 1:** Descriptive Statistics (*n* = 238)

Variable	Summary
Age (years)	64.2 (10.1)^a^
BMI (kg/*m*^2^)	33.1 (5.9)^a^
Pre-operative HgbA1c^b^	6.7(1.0)^a^
Length of Stay (hours)^c^	85.7 (64.4)^a^
Dexamethasone	
Yes	77 (32.4%)
No	161 (67.7%)
Sex	
Male	133 (55.9%)
Female	105 (44.1%)
Type of diabetes	
Type I	12 (5.0%)
Type II	226 (95.0%)
Medication	
Oral Hyperglycemic	119 (50.0%)
Insulin, < 0.6 units/kg/day or 80 units/day	59 (24.8%)
Insulin, > 0.6 units/kg/day or 80 units/day	16 (6.7%)
No medication	44 (18.5%)
Procedure	
THA or Conversion THA	88 (37.0%)
TKA	123 (51.7%)
UKA	27 (11.3%)

^a^Mean (SD) reported

^b^Pre-operative HgbA1c had 19 missing observations

^c^Length of Stay had 2 missing observations

**Table 2:** Patient characteristics by dexamethasone group

Characteristic	Dexamethasone (*N* = 77)		Non-Dexamethasone (*N* = 161)		*P*-value
Age (years)^c^	66.4	(10.8)	63.1	(9.6)	0.021*^d^
BMI ^c^	31.9	(5.6)	33.7	(5.9)	0.031* ^d^
Pre-operative HgbA1c ^c^	6.6	(0.7)	6.8	(1.1)	0.308 ^d^
Length of Stay (hours) ^c^	78.9	(76.7)	89.0	(57.7)	0.065^e^
Sex					
Male	76	(47.2%)	29	(37.7%)	0.165^f^
Female	85	(52.8%)	48	(62.3%)	
Type of diabetes					
Type I	2	(2.6%)	10	(6.2%)	0.233 ^f^
Type II	75	(97.4%)	151	(93.8%)	
Medication					
Oral Hyperglycemic	33	(42.9%)	86	(53.4%)	0.020* ^f^
Insulin A^a^	17	(22.1%)	42	(26.1%)	
Insulin B^b^	4	(5.2%)	12	(7.5%)	
No medication	23	(29.9%)	21	(13.0%)	
Procedure					
THA or Conversion THA	31	(40.3%)	57	(35.4%)	< 0.001*^f^
TKA	25	(32.5%)	98	(60.9%)	
UKA	21	(27.3%)	6	(3.7%)	

^a^< 0.6 units/kg/day or 80 units/day

^b^> 0.6 units/kg/day or 80 units/day

^c^Continuous variables presented as mean ± SD

^d^Two-sample T-test

^e^Wilcoxon Test

^f^Pearson chi-square test

*P-value < 0.05

### Primary outcome

Dexamethasone use was associated with a higher incidence of postoperative hyperglycemia in the first 24 h (*P* <  0.001) and 72 h (P <  0.001) after surgery (Table 3). Specifically, patients who received dexamethasone had 4.03 (95% CI: 2.44, 6.65) and 3.07 (95% CI: 2.34, 4.03) higher odds of post-operative hyperglycemia than those who did not in the first 24 and 72 h respectively (Table 3).

**Table 3:** Adjusted Odds Ratios and Estimates for Primary and Secondary Outcomes

Characteristic	Level	Post-op Hyperglycemia (72 h)		Post-op Hyperglycemia (24 h)	
		Odds Ratio (95% CI)	*P*-value	Odds Ratio (95% CI)	*P*-value
Dexamethasone	Yes – No	3.07 (2.34, 4.03)	< 0.001*	4.03 (2.44, 6.65)	< 0.001*
Use					
Medical	THA – TKA	1.09 (0.83, 1.42)	0.540	1.67 (1.01, 2.76)	0.046*
Procedure	THA – UKA	1.53 (0.93, 2.51)	0.100	1.96 (0.90, 4.33)	0.092
Diabetes type	Type I – Type II	1.89 (1.20, 3.00)	0.006*	0.98 (0.36, 2.68)	0.969
Diabetic	Oral hyperglycemic –	3.89 (2.07, 7.31)	< 0.001*	2.53 (0.97, 6.61)	0.058
Medication	None	7.96 (4.20, 15.09)	< 0.001*	5.37 (1.99, 14.44)	0.001*
	Insulin A^a^– None	10.65 (5.14, 22.10)	< 0.001*	8.77 (2.61, 29.52)	0.001*
	Insulin B^b^– None				
Gender	Female – Male	1.23 (0.94, 1.60)	0.126	1.34 (0.82, 2.20)	0.242
Age	One unit increase	1.01 (1.00, 1.02)	0.226	1.02 (1.00, 1.05)	0.051
BMI	One unit increase	1.00 (0.97, 1.02)	0.615	1.00 (0.96, 1.05)	0.871
Pre-Op HgbA1c	One unit increase	1.38 (1.23, 1.54)	< 0.001*	1.26 (1.03, 1.52)	0.022*
Post-op Hyperglycemia by Dose (72 h)					
Dexamethasone	Yes – No				
Use	4,5, or 8 mg – No	1.75 (1.04, 2.94)	0.034*		
	10 mg – 4,5, or 8 mg	2.01 (1.18, 3.42)	0.010*		
	10 mg – No	3.52 (2.64, 4.71)	< 0.001*		

*P-value < 0.05

^a^< 0.6 units/kg/day or 80 units/day

^b^> 0.6 units/kg/day or 80 units/day

### Secondary outcomes

Furthermore, every one-unit increase in HgbA1c was associated with statistically significant higher odds of post-operative hyperglycemia in the first 24 h (OR = 1.26, 95% CI: 1.03, 1.52, *P* = 0.022) and 72 h (OR = 1.38, 95% CI: 1.23, 1.54, *P* <  0.001) (Table 3).

When comparing diabetic medication regimen to no medications on post-operative hyperglycemia oral medications, insulin less than 0.6 units/kg/day or 80 units/day, and insulin greater than 0.6 units/kg/day all had statistically significant higher odds of postoperative hyperglycemia in the first 72 h after surgery (Table 3). In the first 24 h both doses of insulin had statistically higher odds of hyperglycemia however oral medications did not (Table 3). Age, BMI, and gender were not associated with higher odds of post-operative hyperglycemia.

**Fig. 1:**
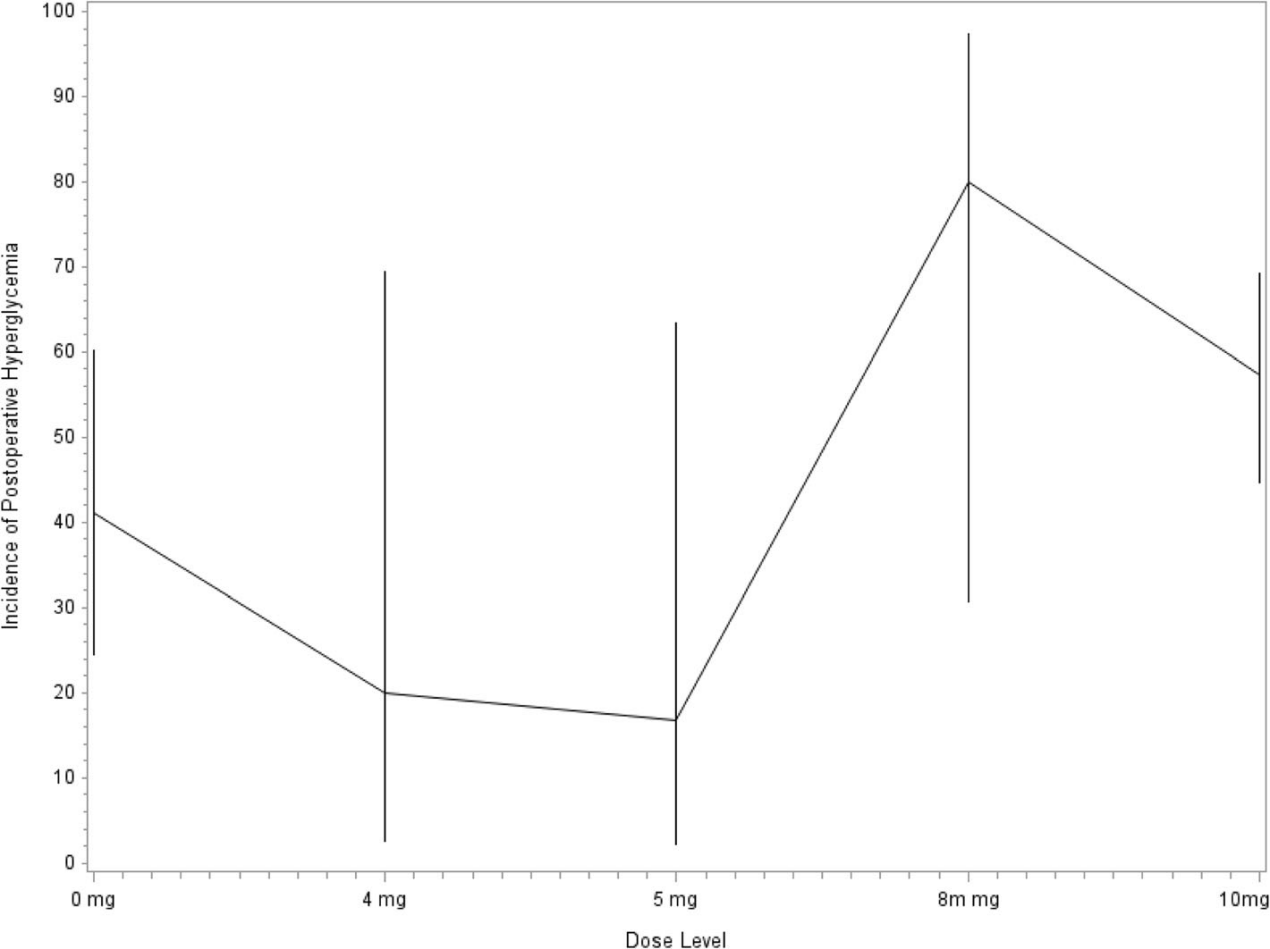
Incidence of postoperative hyperglycemia by dose of dexamethasone

Additionally, the relationship between dose of dexamethasone and post-operative blood glucose was assessed, while controlling for clinical and demographic characteristics. A higher dose of dexamethasone was associated with a higher incidence of blood glucose exceeding 200 mg/dL in the first 72 h after surgery (Fig. 1). Particularly, patients who received 10 mg of dexamethasone had 3.52 (95% CI: 2.64, 4.71; *P*≤0.001) higher odds of any one measurement of post-op blood glucose level being elevated than those who did not receive dexamethasone and had 2.01 (95% CI: 1.18, 3.42; *P*=0.010) higher odds than those who received either 4, 5, or 8 mg (Table 3). Finally, patients who received less than 10 mg of dexamethasone had 1.75 (95% CI: 1.04, 2.94; *P*=0.034) higher odds of an elevated post-op blood glucose measurement than those who did not receive dexamethasone in any dose (Table 3).

**Fig. 2:**
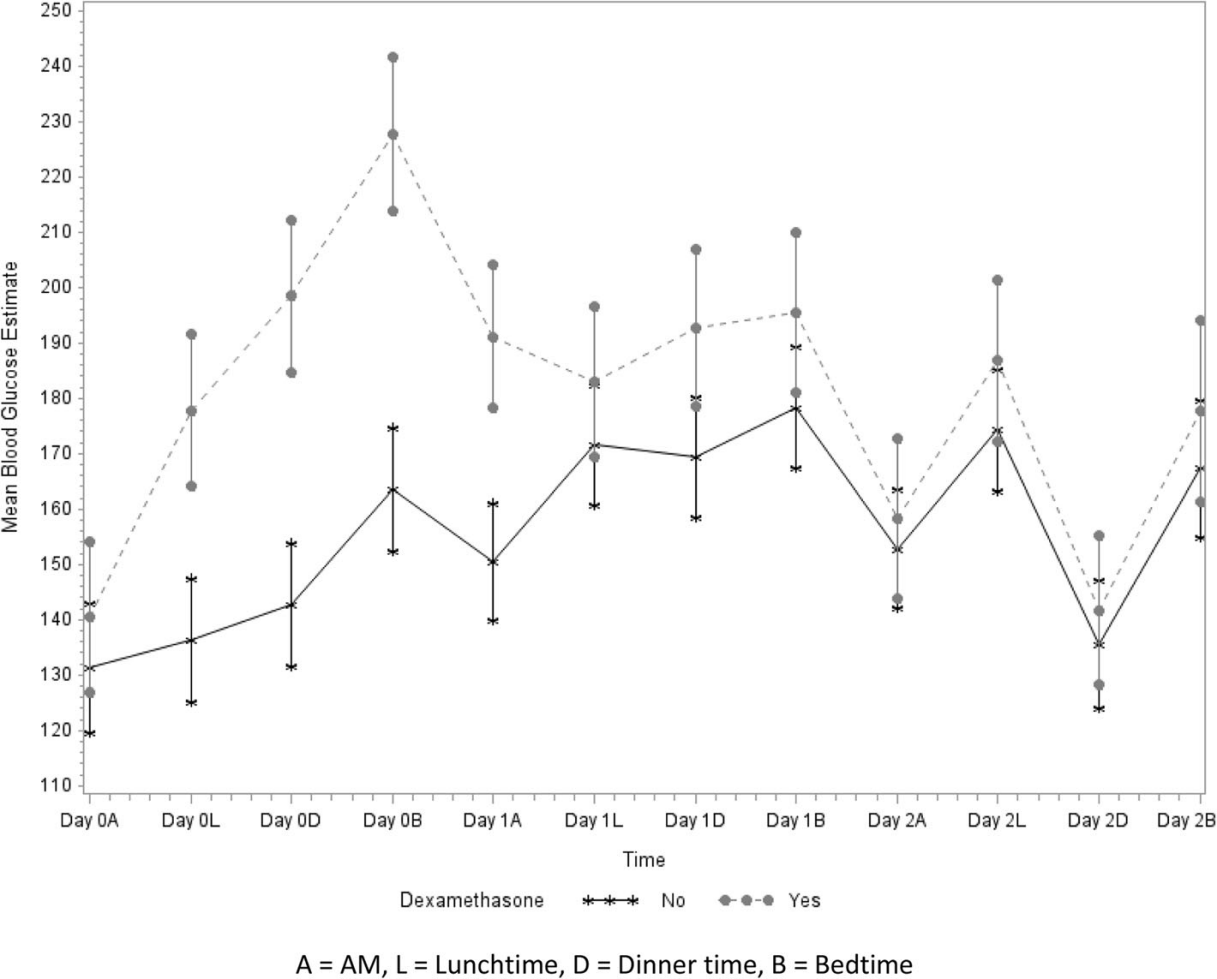
Estimates of mean blood glucose by group over time. A = AM, L = Lunchtime, D = Dinner time, B = Bedtime

Figure 2 shows a plot of the estimates of the mean blood glucose levels by group over time, indicating a significant difference between the dexamethasone group and non-dexamethasone groups across time (*P* <  0.001). The dexamethasone group had higher mean blood glucose levels at Day 0 Lunch (*P*≤0.001), Day 0 Dinner (*P*≤0.001), Day 0 Bedtime (*P*≤0.001), Day 1 AM (*P*≤0.001), Day 1 Dinner (*P* = 0.003) and Day 1 Bedtime (*P* = 0.028).

Finally, there was no statistically significant relationship between dexamethasone use and length of stay (*P* = 0.830).

## Discussion

While dexamethasone has been shown to be effective in reducing postoperative nausea and vomiting, increasing analgesia, decreasing length of stay, and improving patient satisfaction [2–6, 8] there is a concern that the side effect of hyperglycemia could have deleterious outcomes in arthroplasty patients. Our data suggests that in diabetic patients preoperative dexamethasone use increases the risk of postoperative hyperglycemia after primary knee or hip arthroplasty up to 72 h after surgery. While our study did not investigate causation, the data suggests that dexamethasone use in this patient population should be thoughtfully considered.

A large review of 20,171 total hip and knee arthroplasty patients showed a significantly higher risk of prosthetic joint infection (PJI) among patients with a diagnosis of diabetes mellitus, patients using antidiabetic drugs, and patients with perioperative hyperglycemia [9]. Hwang et al. retrospectively reviewed 462 patients who underwent total knee arthroplasty and there was a positive correlation among patients with HgbA1c ≥ 8% and perioperative blood glucose levels ≥200 mg/dL and superficial surgical site infection [12]. Stryker et al. [10] also showed that patients with mean postoperative blood glucose of > 200 mg/dL or a pre-operative HgbA1c level > 6.7% had increased risk for wound complications following elective primary total joint arthroplasty. Finally, in another larger review of 13,272 patients who underwent primary total joint arthroplasty 38% of patients had HgbA1c greater than 7% and that while the HgbA1c did not increase risk of infection, perioperative hyperglycemia was associated with increased incidence of PJI [11]. Therefore, perioperative glycemic control is of significant importance to prevent potentially devastating postoperative complications including PJI or delayed wound healing.

We showed that in the first 72 h after surgery, 41/77 (53.2%) of patients in the study who received dexamethasone had postoperative hyperglycemia. This is in comparison to 62/161 (38.5%) subjects who did not receive dexamethasone. This is much higher than previously reported values by Nurok et al. [14] who showed no significant difference in hyperglycemia between dexamethasone group and non-dexamathasone group with only a 5.6% incidence. However, their study only had a small number of diabetic patients (*n* = 26) with 19/26 not receiving dexamethasone.

In order to study the temporal effects of dexamethasone, Hans et al. analyzed serial blood glucose levels after intravenous dexamethasone administration in diabetic and non-diabetic patients [15]. Blood glucose concentrations increased significantly over time and peaked at 120 min after administration. The maximum concentration of blood glucose was higher in diabetic patients and was associated with increased BMI and HgbA1c. Similarly, in our study, the incidence postoperative hyperglycemia was higher in the first 24 h compared to the first 72 h which is likely attributable to the pharmacokinetics of dexamethasone. This is further supported by the significant increase in blood glucose levels we saw during the first 24 h post-operatively that then normalized by post-operative day two.

In comparison to the study by Backers et al. [3], we were unable to show a significant relationship between length of stay and dexamethasone use. As our population was composed of only diabetic patients, the length of stay could have been attributable to extra time needed to normalize blood glucose post-operatively and confounded by selecting patients with more medical co-morbidities; however our study was not designed to investigate this.

To assess the safety of dexamethasone administration, Richardson et al. performed a retrospective review of 6294 patients investigating the relationship between dexamethasone administration and infection rates after primary arthroplasty. They found no difference in PJI requiring surgical intervention after a single dose of perioperative dexamethasone [7]. However there were only 557 patients that received dexamethasone versus 5737 that did not. They were also unable to identify transient postoperative hyperglycemia or cases of delayed wound healing due to study design flaws.

There were several inherent limitations to this study. As a retrospective review we were not able to control for dexamethasone administration or dose. At our institution one provider routinely gives dexamethasone to all patients while others use it selectively in non-diabetic patients or patients with well-controlled diabetes. This likely induced selection bias as indicated by the differences in our study groups. Patients who did not get dexamethasone tended to have higher BMI’s and increased anti-diabetic medication requirements pre-operatively (Table 2). While this is a clear source of bias, it only potentially underestimated the true effect of dexamethasone in patients with more severe diabetes. At the same time, there was no morning day of surgery blood glucose check or standard glucose control protocols. While there was no significant difference between hemoglobin A1c levels between the dexamethasone group and non-dexamethasone group, we are unable to say with certainty that the blood sugar effect that was seen was not related to morning of surgery hyperglycemia or glucose control protocols. However, we were able to show a dose response, which supports the relationship between dexamethasone and post-operative hyperglycemia. Furthermore, there were missing data points for blood glucose concentrations and we therefore could have missed episodes of hyperglycemia. However all patients had a morning blood glucose level on postoperative days one and two and had at least one post-prandial measurement each day therefore our data sample was likely representative of all values. Clearly these limitations could be addressed with a prospective randomized study in diabetic and non-diabetic patients to determine if dexamethasone is an independent risk factor for hyperglycemia. However, to the best of our knowledge, our study is the only one that evaluates the effects of dexamethasone specifically in a diabetic patient population after primary hip or knee arthroplasty.

## Conclusions

While a low dose of perioperative dexamethasone (10 mg or less) has been shown to be a valuable part of a comprehensive multimodal analgesic regimen in low-risk arthroplasty patients, its use in diabetic patients should be thoughtfully considered. While it may be effective in mitigating nausea, vomiting, analgesia, and reducing hospital length of stay our data shows that there is significant increased risk for perioperative hyperglycemia in diabetic patients who receive dexamethasone. Given the known impact of in-hospital hyperglycemia on infection and other complications in surgical patients, physicians should consider patient characteristics to guide safe use of perioperative dexamethasone. Further prospective studies are needed to determine the long-term effects of dexamethasone in diabetic patients as it relates to complications such as PJI.

## Abbreviations

BMI: Body Mass Index
CI: Confidence Interval
CPT: Current Procedural Terminology
HgbA1c: Hemoglobin A1c
LOS: Length of Stay
OR: Odds Ratio
PJI: Prosthetic Joint Infection
THA: Total Hip Arthroplasty
TKA: Total Knee Arthroplasty
UKA: Unicondylar Knee Arthroplasty
